# Fe_3_O_4_/Graphene Composite Anode Material for Fast-Charging Li-Ion Batteries

**DOI:** 10.3390/molecules26144316

**Published:** 2021-07-16

**Authors:** Antunes Staffolani, Hamideh Darjazi, Gilberto Carbonari, Fabio Maroni, Serena Gabrielli, Francesco Nobili

**Affiliations:** Chemistry Division, School of Science and Technology, University of Camerino, 62032 Camerino, Italy; antunes.staffolani@unicam.it (A.S.); hamideh.darjazi@unicam.it (H.D.); gilberto.carbonari@unicam.it (G.C.); fabio.maroni@unicam.it (F.M.); serena.gabrielli@unicam.it (S.G.)

**Keywords:** Li-ion batteries, conversion materials, graphene, anode materials, electrochemical impedance spectroscopy

## Abstract

Composite anode material based on Fe_3_O_4_ and reduced graphene oxide is prepared by base-catalysed co-precipitation and sonochemical dispersion. Structural and morphological characterizations demonstrate an effective and homogeneous embedding of Fe_3_O_4_ nanoparticles in the carbonaceous matrix. Electrochemical characterization highlights specific capacities higher than 1000 mAh g^−1^ at 1C, while a capacity of 980 mAhg^−1^ is retained at 4C, with outstanding cycling stability. These results demonstrate a synergistic effect by nanosize morphology of Fe_3_O_4_ and inter-particle conductivity of graphene nanosheets, which also contribute to enhancing the mechanical and cycling stability of the electrode. The outstanding capacity delivered at high rates suggests a possible application of the anode material for high-power systems.

## 1. Introduction

Current concerns about limited energy resources, coupled with the need to decrease greenhouse gas emissions, are leading worldwide research and development efforts to enhance efficient utilization of renewable energies as main energy sources.

Due to the fitful nature of renewable sources’ availability and energy needs, efficient energy storage for stationary and mobile applications is mandatory. Among the available technologies, Li-ion batteries have played a main role for the last three decades, due to their current performances and development prospects. However, to fulfil the needs of emerging and consolidating technologies, such as electrified vehicles, the research activity has had to focus efforts on new materials with improved power and energy densities.

On the anode side, insertion materials represent the state-of-art, especially graphite [1], thanks to its unique features, such as flat and low working potential vs. lithium, low cost, and good cycle life. However, graphite allows the intercalation of a limited number of lithium ions dictated by its layered structure, leading to a stoichiometry of LiC_6_ as the end-term of intercalation process and a specific capacity limited to 372 mAh g^−1^ [2].

In order to overcome these drawbacks, research has focused on different reactivity concepts, such as the conversion process, entailing the reversible electrochemical reaction of lithium with transition metal oxide (TMO). The conversion reaction can be generalized as in Equation (1):

(1) $$M_{a}O_{b}+2{\mathrm{bLi}}^{+}+2{\mathrm{be}}^{-}↔\mathrm{aM}+{\mathrm{bLi}}_{2}O$$

where M is a transition metal such as Ni, Mn, Fe, etc.

Several TMOs have been studied as possible candidate anodes for LIBs [3], such as Co_3_O_4_ [4], Fe_2_O_3_ [5] and Fe_3_O_4_ [6]. Among them, the very large capacities associated with the conversion of iron oxides, coupled with their low toxicity and cost, make them attractive candidate for high-capacity batteries.

However, the conversion mechanism usually suffers from a series of issues intimately connected with the conversion reaction itself. Indeed, remarkable structural change and volume expansion are associated with the conversion process [7], eventually leading to pulverization and detachment from the current collector.

In order to improve the electrochemical behavior, several practical approaches have been considered: the use of composite nanoarchitectures and optimized nanomorphologies, such as nanorods [8], hollow [9] or nanosphere [10], carbon coating [11], nanopowder [12]; as a result, graphene-based composites have shown remarkable improvements [13].

Graphene has drawn much attention in material science thanks to its remarkable properties, such as reliable thermal conductivity [14], good electrical conductivity [15], and superior mechanical properties [16]. However, its ability to serve as an active anode material for LIBs is severely questioned [17]. In fact, despite its high theoretical capacity of 744 mAh g^−1^, due to the ability to store Li on both sides of isolated graphene layers, very quickly the layers tend to restack to form the thermodynamically more stable amorphous carbon and, eventually, graphite, thus negating long-term advantages over more ‘classical’ layered carbon structures.

Nevertheless, thanks to its favorable electrical and mechanical properties, graphene is a promising embedding matrix, which could improve the mechanical resistance of electrodes based on active materials suffering of large volume changes and mechanical instability, such as the conversion-based ones. In addition, the overall electrodes conductivity may be enhanced. As a consequence of this, several nanocomposites based on graphene and transition metals [18] or transition metal oxides [19,20,21] have been studied as promising anode materials for LIBs.

Despite its inactive role, the polymeric binder is essential for the electrode manufacturing and, more importantly, it defines the cost and environmental impact of the battery pack. Indeed, the state-of-the-art PVdF, a highly-fluorinated polymer is not only expensive but also requires a toxic and expensive solvent/dispersant, i.e., *N*-methyl-2-pyrrolidone (NMP) for electrode processing. In this regard, polyacrylic acid (PAA), a polymer belonging to the family of polyacrylates has already proven its advantages when used for Si- and conversion oxide-based electrodes [6,22,23,24]. Furthermore, it is soluble/dispersible in cheap environmentally friendly media, such as water or ethanol.

Herein, we report a facile one-pot synthesis of Fe_3_O_4_ nanoparticles by coprecipitation of FeCl_2_∙4H_2_O and FeCl_3_∙6H_2_O in the presence of NH_4_OH, and their embedding in a reduced graphene oxide (Fe_3_O_4_/rGO) matrix prepared by ultrasonication of graphene oxide and subsequent reduction with hydrazine. Anodes are then prepared by using Polyacrylic acid (PAA) as an a high-elastic-modulus binder [23], with the aim to mitigate the lithiation/delithiation mechanical stresses and guarantee a low environmental impact of electrode fabrication.

## 2. Results

### 2.1. Structural and Morphological Characterization

Raman spectroscopy was applied to characterize the chemical structure of Fe_3_O_4_/reduced graphene oxide composite; furthermore, bare Fe_3_O_4_ nps and rGO were analysed as reference materials.

**Figure 1:**
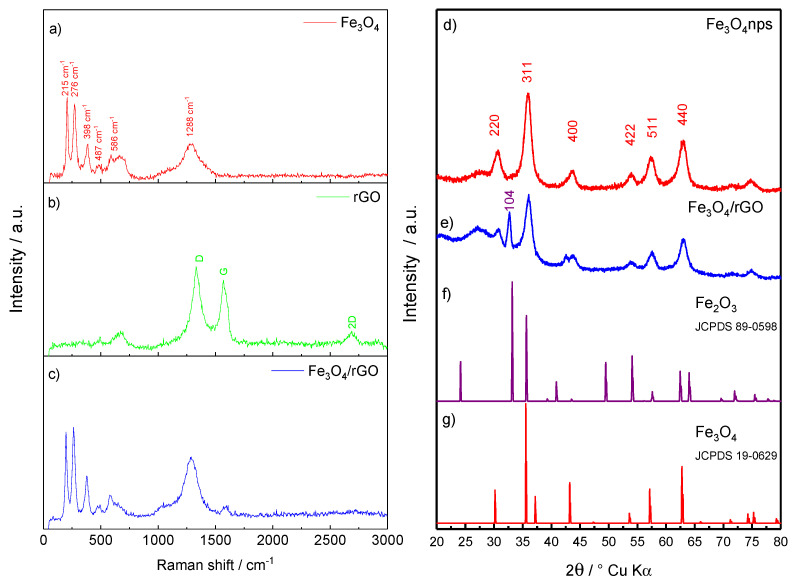
(**a**) Raman spectra of pristine Fe_3_O_4_ nanoparticles, (**b**) rGO (red line), and (**c**) Fe_3_O_4_/rGO. X-ray diffraction patterns of (**d**) Fe_3_O_4_ nanoparticles and (**e**) Fe_3_O_4_/rGO; for reference, the JCPDS card of (**f**) Fe_2_O_3_ (89-0598) and (**g**) Fe_3_O_4_ (19-0629).

The Raman response of bare Fe_3_O_4_ nanoparticles is shown in Figure 1a. A series of peaks, which are consistent with literature findings, is revealed [25,26]. The peaks located at 276, 398, 487, and 586 cm^−1^ can be ascribed to the characteristic vibration modes of Fe-O bonds [25,26]. The D-band located at 1288 cm^−^^1^ can be indexed to the defects present on the surface of the nanoparticles. In addition, the presence of Fe_2_O_3_ additional phase is revealed by the peaks at 215 and 276 cm^−1^. Even if Fe_2_O_3_ is expected only in minor amounts, the relatively strong signal is probably due to an enhancement, which can be ascribed to a partial oxidation of Fe^2+^ to Fe^3+^ occurring by laser irradiation during the Raman measurement [27].

Figure 1b shows the Raman response of bare rGO. Three characteristic peaks of carbonaceous materials are observed [28]. The D band located at 1338 cm^−1^ and the G band located at 1581 cm^−1^ shows a recovery of the hexagonal pattern of carbon atoms with defects. The high ID/IG ratio (≈1.31) evidence a high number of structural defects on rGO surface.

Figure 1c shows the Raman response of the Fe_3_O_4_/rGO nanocomposite. All the signals evidenced in Figure 1a,b are retained, suggesting that the Fe_3_O_4_ embedding by the rGO matrix relies on electrostatic interactions, without any chemical modifications.

The diffractogram of the pristine magnetite powder (Figure 1d) presents a series of reflections consistent with Fe_3_O_4_ as indexed in JCPDS, card no. 19-0629. The crystallite size was estimated to be ~5.65 nm, by applying the Scherrer’s equation (Equation (2)) [29]:

(2) $$L=\frac{k\lambda }{\beta cos\theta }$$

with k = 0.94, λ = 0.709319 Å (Mo Kα source) and β = FWHM (full-width at half-maximum), the 311 reflection has been considered for the calculation.

The X-ray diffractogram of the magnetite-carbon composite (Figure 1e) still shows the Fe_3_O_4_ XRD reflections 220, 311, 400, 422, 511, 440. A reflection given to Fe_2_O_3_ impurities, corresponding to the set of 104 planes, is also visible, while the other reflections of the Fe_2_O_3_ XRD pattern are probably overlapped by Fe_3_O_4_. By applying the Scherrer equation, the crystallite size can be estimated to ~5.80 nm. In addition, a large hump around 2*θ* = 26° is the signature of the amorphous rGO carbon phase. Figure 1f,g show the theoretical reflections of the Fe_3_O_4_ and Fe_2_O_3_ phases.

**Figure 2:**
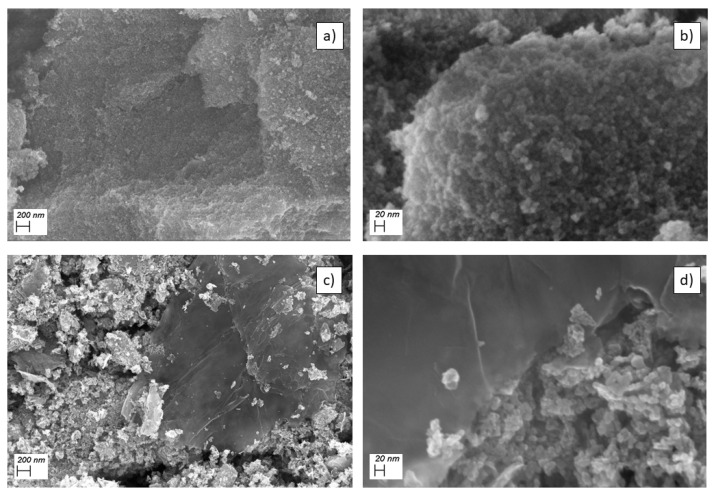
SEM micrographs of pristine Fe_3_O_4_ nanoparticles (**a**) at 40,000× and (**b**) at 275,000× magnification levels. SEM micrographs of Fe_3_O_4_/rGO (**c**) at 40,000× and (**d**) at 275,000× magnification levels.

Figure 2a,b show the SEM micrographs of Fe_3_O_4_ nanoparticles at 40,000× and 275,000× magnification levels, respectively. Despite the formation of aggregates, probably due to agglomeration by magnetic stirring, the particle size can be visually estimated to 5–10 nm, consistent with the crystallite size estimation.

Figure 2c,d show the SEM micrographs of Fe_3_O_4_/rGO nanocomposite at 40,000× and 275,000× magnification levels, respectively. In both SEM images, carbon sheets embedding nanoparticles bigger than the pristine oxide are clearly evidenced, suggesting a probable agglomeration during the composite synthesis. At the higher magnification, it is possible to see iron oxide nanoparticles embedded into the amorphous carbon sheets.

### 2.2. Thermal Characterization

**Figure 3:**
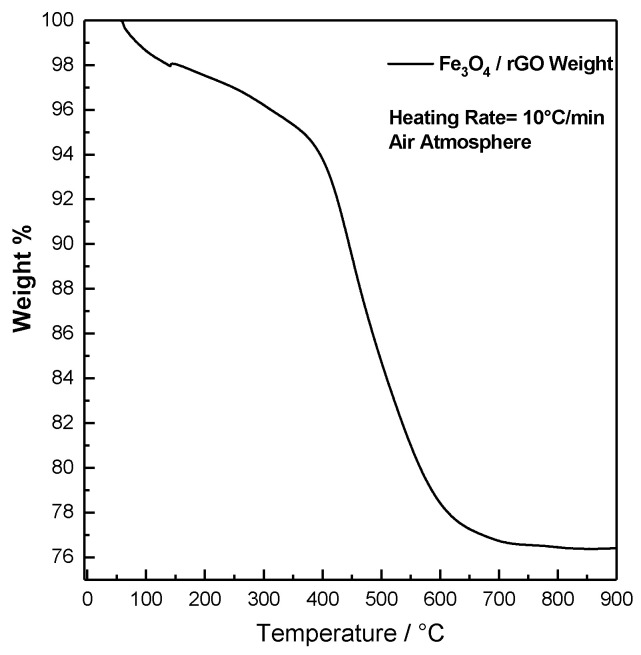
Thermogravimetric characterization of Fe_3_O_4_/rGO at 10°/min in air atmosphere.

Figure 3 depicts the thermogravimetric analysis of Fe_3_O_4_/rGO.

The composite was heated with a heating rate of 10 °C/min in air atmosphere. After some low-T weight loss due to water evaporation, the analysis shows a weight drop from around 96% to 76% (about 20%) at T ≈ 450 °C, due to the oxidation of the rGO to carbon dioxide. This corresponds to an approximate Fe_3_O_4_:rGO mass ratio of 79:21, which allows to estimate specific capacity for the composite material of the order of 885 mAh g^−1^. This section may be divided by subheadings. 

### 2.3. Electrochemical Characterization 

**Figure 4:**
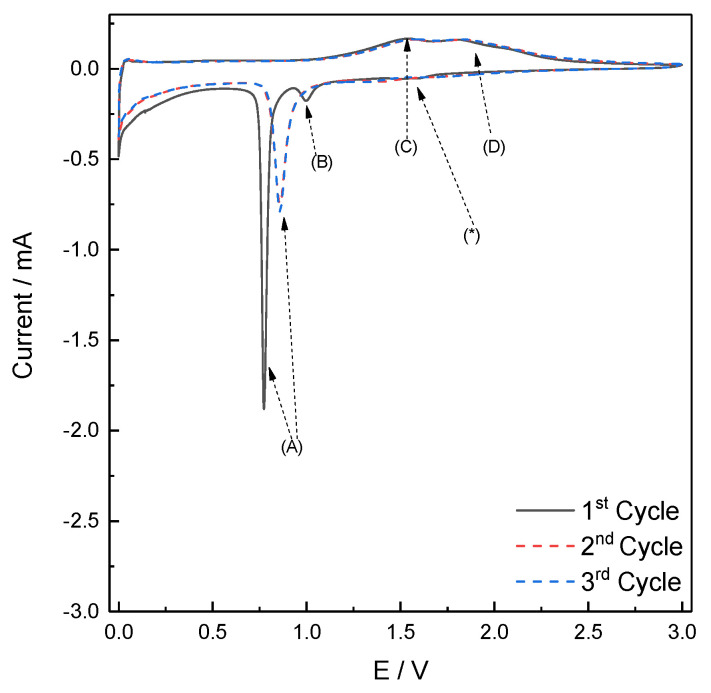
Cyclic voltammetry of Fe_3_O_4_/rGO performed in the potential window within 0.001 < E < 3.000 V vs. Li^+^/Li; scan rate = 0.1 mV s^−1^.

As shown in Figure 4, the voltametric response of Fe_3_O_4_/rGO composite material reveals, during the first cathodic scan, three main features: a very broad, and low hump around 1.5 V (*), and two peaks at 1.02 V (B) and 0.75 V (A). The feature around 1.5 V (*) has been observed also for other transition metal oxides [30], and describes irreversible interfacial processes only occurring during the first discharge.

As regards the peak at 1.02 V (B), Thackeray [31] proposes a mechanism in which intercalation of Lithium into the spinel structure of Fe_3_O_4_ occurs according to Equation (3):

(3) $${\;\mathrm{Fe}}_{3}O_{4}+2{\mathrm{Li}}^{+}+2e^{-}\to {\mathrm{Li}}_{2}{\mathrm{Fe}}_{3}O_{4}$$

The subsequent reduction of Fe^3+^ to Fe^0^ (described with the sharp peak at 0.75 V (A)) occurs by the conversion reaction leading to the Fe^0^ nanoparticles dispersed in a Li_2_O matrix (Equation (4)):

(4) $${\;\mathrm{Li}}_{2}{\mathrm{Fe}}_{3}O_{4}+6{\mathrm{Li}}^{+}+6e^{-}\to 3{\mathrm{Fe}}^{0}+4{\mathrm{Li}}_{2}O$$

In this potential region, the decomposition of electrolyte towards the carbon surface, forming the passivation layer, take place. During the anodic scan, a couple of broad peaks (C) and (D) are visible at 1.57 V and 1.89 V and can attributed to the oxidation of Fe^0^ to Fe^2+^ and Fe^3+^ respectively.

During the cathodic scans of the subsequent cycles, peak (B) disappears and peak (A) is shifted to 0.8 V, while the behavior during anodic scans is retained.

**Figure 5:**
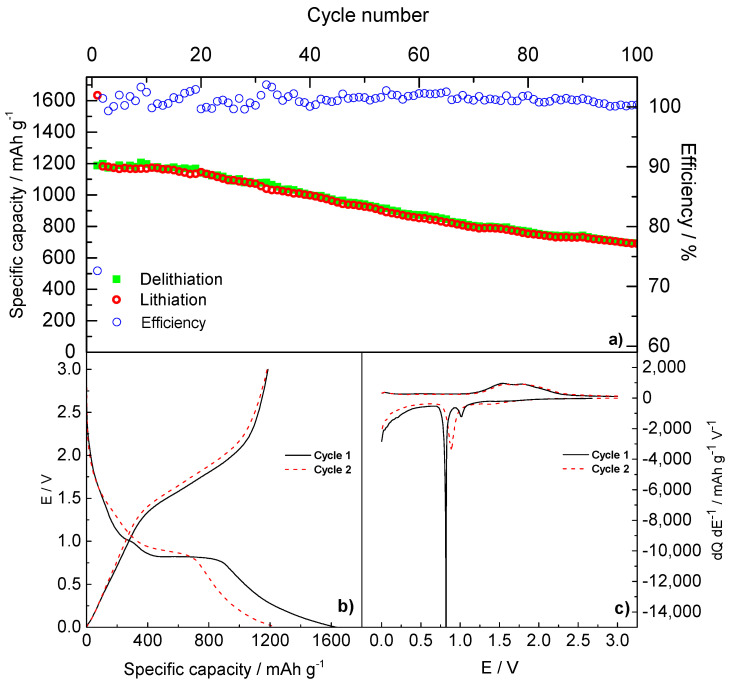
(**a**) Galvanostatic cycles at 1C-rate of Fe_3_O_4_/rGO, (**b**) galvanostatic E vs. Q profile of the first 2 cycles, (**c**) differential dQ dE^−1^ profile. The test was performed at 1C-rate (881 mA g^−1^) and in the potential window within 0.001–3.000 V.

Figure 5 shows the galvanostatic cycling behavior of Fe_3_O_4_/rGO composite anode at the estimated 1C rate.

The specific capacity of the first discharge reaches a value of 1634 mAh g^−1^, while the subsequent charge exhibits a value of 1186 mAh g^−1^, with an efficiency of 72%. During the following 40 cycles, both lithiation and delithiation specific capacities remain higher than 1000 mAh g^−1^, with an efficiency close to 100%. Capacity values higher than the theoretical ones have already been reported for conversion materials by several authors and could be associated with several mechanisms. Among those, interfacial lithium storage [32] and reversible Li storage processes by the external layers of SEI, mainly given by the partly reversible formation/dissolution of carbonates and semicarbonates [33] and commonly described as a ‘gel-type layer’ [34], can provide extra capacity. 

Capacity subsequently fades upon long-term cycling, with a drop to around 700 mAh g^−1^ at the 100th cycle, still with a coulombic efficiency close to 100%.

The galvanostatic E vs. Q profiles (Figure 5b) reveal during the first discharge a shoulder around 1.5 V, a small plateau at 1.01 V, and a larger one at 0.81 V for the lithium insertion and conversion reactions, while the charge step shows a slopping plateau from 1.54 V to 1.83 V due to the oxidation of Fe. These plateaus are consistent with the differential analysis dQ dE^−1^ vs. E (Figure 5c), which reveals, during the first lithiation, a broad hump around 1.5 V (*), a peak (A) at 0.80 V and a peak (B) at 1.01 V, while peaks (C) and (D) at 1.54 V and 1.80 V, corresponding to the subsequent Fe oxidation steps, are observed during delithiation. During the second lithiation/delithiation cycle, any sign of the previous plateaus disappears in favor of a more pronounced plateau at 0.89 V. The curves are characterized by a large voltage hysteresis, which is a typical feature of conversion materials. This phenomenon has been explained by several authors by concurring processes, such as a pseudocapacitive behavior due to the increase of surface area upon cycling [35], or different reaction pathways for conversion and deconversion reactions [36].

**Figure 6:**
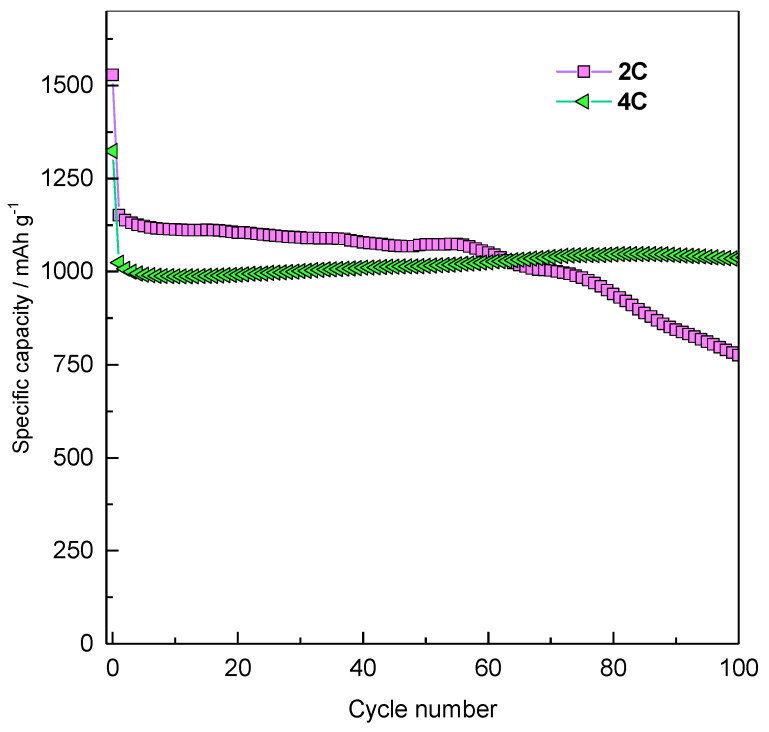
Fe_3_O_4_/rGO performances at 2C and 4C rate. The tests were performed at 1762 and 3524 mA g^−1^ (2C and 4C, respectively), in the potential window within 0.001–3.000 V.

Figure 6 reports the performances of the Fe_3_O_4_/rGO composite material at 2C (1848 mA g^−1^) and 4C rate (3696 mA g^−1^).

The material shows very stable performances at high rates, thanks to graphene and its high electronic conductivity. The first discharge at 2C gives a capacity of 1529 mAh g^−1^, and the subsequent charge delivers 1103 mAh g^−1^, with an initial coulombic efficiency of 72%. The capacity remains quite stable until cycle 55, giving an average value of 1050 mAh g^−1^. At 4C the material exhibits a first-cycle discharge capacity of 1323 mAh g^−1^ and a charge capacity of 993 mAh g^−1^, with an initial coulombic efficiency of 75%. Also at this high C-rate, the charge/discharge capacity remains quite stable during the cycling, with an average value of 980 mAh g^−1^, suggesting that the Fe_3_O_4_/rGO composite works better at high rates. The difference in performances and stability between the lower (1C) and higher (2C, 4C) rates may be rooted in a possible decomposition of the electrode material at lower rates, due to irreversible chemical processes concurrent with the electrochemical Li storage. Furthermore, the SEI formation and irreversible processes at slow rate are enhanced by the high surface area of graphene sheet. On the contrary, as the rate increases, the reversible electrochemical processes become predominant and the performances of the electrode are very stable.

**Figure 7:**
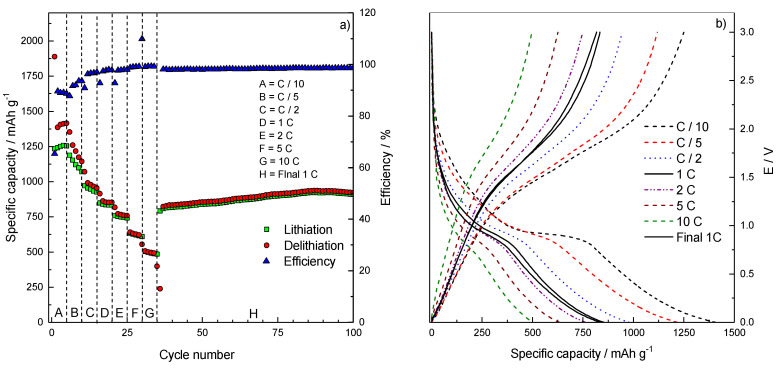
(**a**) Rate capability of Fe_3_O_4_/rGO performed in the potential window within 0.001 < E < 3.000 V vs. Li^+^/Li. (**b**) Galvanostatic E vs. Q profiles of third cycle at each rate.

These findings have been validated by rate capability measurements in an extended C-range, from C/10 (88 mA g^−1^) up to 10C (8800 mA g^−1^). The rate capability results of Fe_3_O_4_/rGO are presented in Figure 7 and detailed in Table 1.

The rate capability test confirms that the charge/discharge behavior of Fe_3_O_4_/rGO (Figure 7a) is more stable at higher than at lower currents, supporting the hypothesis that the low-current high irreversible capacity is rooted in irreversible processes that are enhanced at low C-rates. In fact, up to C/2 some capacity fade is evidenced, which can be ascribed to irreversible, slow-kinetics interfacial phenomena, as confirmed by relatively low coulombic efficiencies.

As the rate increases, the specific capacity decreases, reaching the minimum of 484 mAh g^−1^ at 10C with a coulombic efficiency of 99.2%. When the 1C charge/discharge rate conditions are restored, the electrode is able to retain a steady reversible capacity of ~900 mAh g^−1^. The increase of capacity during the initial cycles at this regime can be ascribed to the progressive electrode morphological rearrangement and wetting by electrolyte, which makes more active sites accessible by Li^+^ ions.

When the Fe_3_O_4_/rGO results here shown are compared with those reported in Ref. [6] by a pristine Fe_3_O_4_ anode prepared in the same conditions, some similarities are evidenced. In fact, at the lower C-rate values (C/10, C/5, C/2), irreversibility and capacity fades are evidenced for both electrodes by the relatively low coulombic efficiencies (91.4% vs. 88.6% for Fe_3_O_4_ and Fe_3_O_4_/rGO at C/10, respectively). However, the current composite Fe_3_O_4_/rGO electrode constantly shows capacity values, which are about 150 mAh g^−1^ higher than pristine Fe_3_O_4_ at C-rate > C/2. Also at higher rates, when both electrodes exhibit enhanced capacity retention, the present Fe_3_O_4_/rGO composite exhibits both better coulombic efficiency and higher capacity than the pristine Fe_3_O_4_. The difference is strongly evidenced at the highest rate (10C) with the Fe_3_O_4_/rGO composite exhibiting 484 mAh g^−1^ capacity vs. 293 mAh g^−1^ of the benchmark Fe_3_O_4_. This behavior may be explained by considering that the rGO matrix, in addition to better buffering and confining the volume changes and structure rearrangements that take place upon reversible Li storage, also strongly enhance electrode conductivity, possibly resulting in lower overall electrode polarization and better high-rate tolerance. The profiles of E vs. Q galvanostatic cycles selected at every C-rate Figure 7b are consistent with the evidenced charge/discharge performances: after the 1st cycle, where most of the irreversible processes take place, charge and discharge capacities are always matching, confirming the high coulombic efficiency in every of the cycling conditions investigated. In addition, the low polarization is confirmed by the fact that the lithiation/delithiation plateaus, around 1 V and 1.5 V, respectively, are still visible even at the highest current applied (10C). Comparison of low- and high-C-rate performances with some literature findings is briefly evidenced in Table 2.

With the aim to shed light onto Li exchange kinetics, with particular regard to the role played by the rGO matrix, the impedance response of both electrodes, based on bare Fe_3_O_4_ and Fe_3_O_4_/rGO composite, has been characterized. 

**Figure 8:**
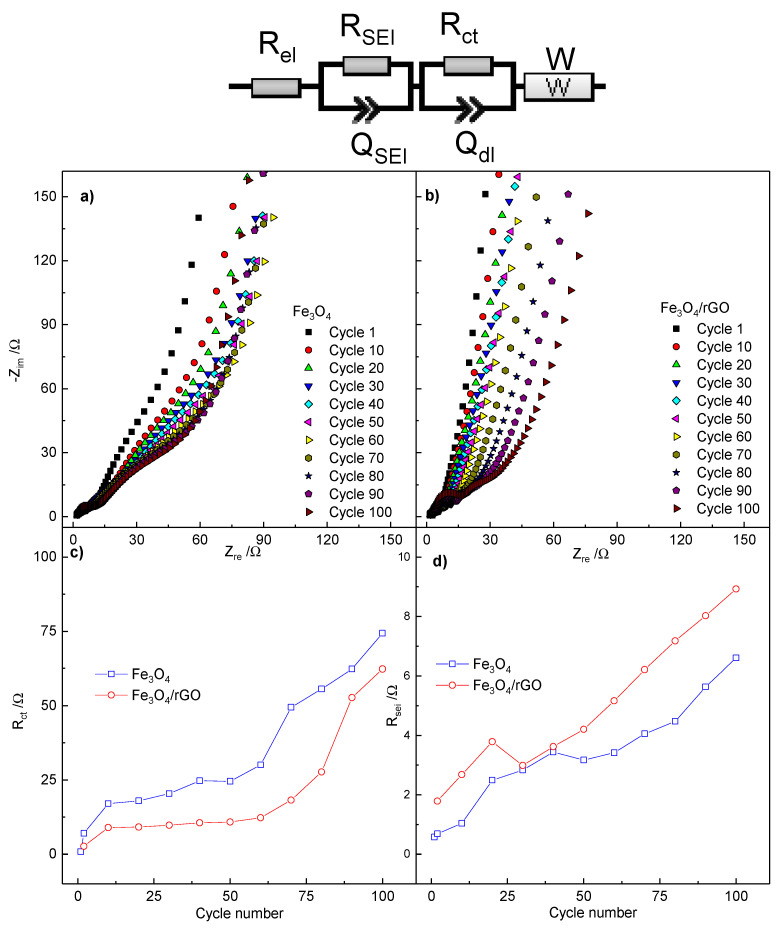
(**a**) Nyquist plot of Fe_3_O_4_ nps acquired every 10 cycles, (**b**) Nyquist plot of Fe_3_O_4_/rGO acquired every 10 cycles. (**c**) Evolution R_SEI_ for both materials upon cycling and (**d**) evolution of R_ct_ upon cycling. The PEIS measurement were conducted in the frequency range of 101 KHz < f < 9 mHz at 0.75 V in the first cycle and at 0.9 V for the further ones, with a sinusoidal perturbation of 5 mV.

Figure 8a,b shows the Nyquist plots acquired at selected cycles (first cycle, then every tenth cycles) during lithiation of Fe_3_O_4_ and Fe_3_O_4_/rGO-based electrodes, respectively. The applied bias potentials correspond to the voltametric peak (A) in Figure 4, i.e., E = 0.75 V for the first cycle and 0.9 V for the following cycles. All the impedance dispersions reveal common features, which are typical for a lithium-ion battery anode [6,41,42], namely: (i) an intercept on the real axis corresponding to electrolyte resistance; (ii) a high-frequency arc corresponding to charge accumulation and migration through the passivation layer, partially overlapped by (iii) a medium-frequency arc corresponding to interfacial charge transfer and electrical double layer formation; (iv) a 45° dispersion, bending toward a vertical line, corresponding to diffusion to a blocking electrode.

For both electrodes, as expected, the overall impedance increases with cycling. The most relevant contribution to the growth is given by the medium-frequency arc related to the charge-transfer, which can be explained either by possible particle aggregation or by increase of inter-particle resistance. A minor increase is evidenced by the high-frequency arc, which may be associated with a limited growth of passivation layer upon cycling. 

When the impedance responses of bare Fe_3_O_4_ and Fe_3_O_4_/rGO are compared, it appears that for the rGO-modified anode, the overall increase is more limited, but the growth of the high-frequency arc is more marked, probably because of a larger electrode/electrolyte interface provided by the carbonaceous rGO matrix. At the same time, for the rGO-modified anode, the medium-frequency arc exhibits a limited growth upon cycling, confirming the dual role of graphene matrix in stabilizing the electrode morphology and in enhancing electrode conductivity and rate of charge-transfer process. The ac-dispersions have been modeled, by using Boukamp’s EQVCRT software [43], to an equivalent circuit R_el_(R_SEI_C_SEI_)(R_ct_C_dl_)W in Boukamp’s notation. R_el_, R_SEI_, C_SEI_, R_ct_, C_dl_ and W represent the pure ohmic resistance of the electrolyte, the resistance and capacitance associated with the passivation layer, the resistance of the charge-transfer process, the capacitance of the electrical double layer, and the Warburg diffusion element, respectively. During the fit procedure, constant phase elements Q substituted pure capacitors C in order to take into account electrode inhomogeneities and roughness [44]. The evolutions of R_ct_ and R_SEI_ upon cycling are reported in Figure 8c,d, respectively. For both materials, the calculated values of both resistances increase upon cycling. Specifically, the Fe_3_O_4_/rGO nanocomposite exhibits a lower R_ct_ than the pristine Fe_3_O_4_, which confirms the enhanced electronic conductivity of the composite, given by the rGO matrix. On the contrary, the R_SEI_ is higher in the composite, which confirms the role played by the large surface area of rGO nanosheets in the buildup of a more extended passivation layer.

## 3. Materials and Methods

### 3.1. Materials

FeCl_2_∙4H_2_O, FeCl_3_∙6H_2_O, hydrazine hydrate and concentrated NH_4_OH were purchased from Sigma-Aldrich (Schnelldorf, Germany) and used as received. Graphene oxide (C:O ratio of 5:4.3) was purchased from Nanoinnova technologies SL (Illescas, Spain) and used as received.

### 3.2. Synthesis

A base promoted coprecipitation of FeCl_2_ and FeCl_3_ was pursued for the synthesis of pristine Fe_3_O_4_ nanoparticles [6]. The synthesis of Fe_3_O_4_ in basic media without any thermal annealing treatment have been widely investigated [45]. The formation mechanism has been explained by the following set of reactions (Equations (5)–(9)):

(5) $${\mathrm{Fe}}^{3+}+3{\mathrm{OH}}^{-}\to \mathrm{Fe}{\left(\mathrm{OH}\right)}_{3\left(s\right)}↓$$

(6) $$\mathrm{Fe}{\left(\mathrm{OH}\right)}_{3}\to {\mathrm{FeOOH}}_{\left(s\right)}+H_{2}O$$

(7) $${\mathrm{Fe}}^{2+}+2{\mathrm{OH}}^{-}\to \mathrm{Fe}{\left(\mathrm{OH}\right)}_{2\left(s\right)}↓$$

(8) $$2{\mathrm{FeOOH}}_{\left(s\right)}+\mathrm{Fe}{\left(\mathrm{OH}\right)}_{2\left(s\right)}\to {\mathrm{Fe}}_{3}O_{4\left(s\right)}+2H_{2}O$$

With the overall reaction:

(9) $$2{\mathrm{FeCl}}_{3}+{\mathrm{FeCl}}_{2}+8{\mathrm{NH}}_{4}\mathrm{OH}\to {\mathrm{Fe}}_{3}O_{4\left(s\right)}+4H_{2}O+8{\mathrm{NH}}_{4}\mathrm{Cl}$$

In this regard, stoichiometric amounts of FeCl_2_∙4H_2_O and FeCl_3_∙6H_2_O were dissolved in 40 mL of distilled water. After the complete dissolution of the salts, 100 mL of 10% NH_4_OH was added to the solution giving a black precipitate. The solution was stirred and heated until reaching 70 °C and then further 30 mL of concentrated NH_4_OH were added and left to react for 8 h. The obtained black powder was easily washed first with acetone and then with ethanol. After that, it was vacuum dried at 50 °C.

200 mg of graphene oxide was added to 150 mL of distilled water and sonicated for 1 in order to obtain a homogeneous suspension. After the sonication, 500 mg of pristine Fe_3_O_4_ nanoparticles were added to the solution and sonicated again for 1 h. After the second sonication, 10 mL of hydrazine hydrate was added to the solution and an ice bath was used to dissipate reaction heat. The solution underwent a third sonication for 2 h. The obtained powder was filtered with millipore (0.2 µm GTTP), thoroughly washed with ethanol, and vacuum dried at 50 °C.

### 3.3. Electrode Processing 

Electrodes based both on the Fe_3_O_4_/rGO composite were prepared by using the same procedure. The anode slurries were obtained by mixing active material, SuperC65 (TIMCAL C-ENERGY TM) as a conductive agent and Polyacrilic Acid (Mw = 450,000—Aldrich) as a binder in the ratio 70:20:10, respectively. The active material and the conductive agent were finely grounded in an agata mortar and then added to the binder solution. The slurries were prepared using ethanol as solvent and stirred by a magnetic stirrer overnight. The well-mixed slurries were first casted on copper foil by Doctor Blade at 100 µm wet thickness and dried on a heating plate at 70 °C for 2 h. Circular electrodes (9 mm diameter) were cut using an electrode puncher (EL-CELL). Electrodes were further pressed using a hydraulic press at 4.7 tons cm^−2^. The average loading of active material was of ≈1 mg cm^−2^. Eventually, the electrodes were dried at 120 °C under vacuum for 12 h and stored in an Ar-filled glovebox.

### 3.4. Structural and Electrochemical Characterizaiton

The structure of the prepared materials was characterized by Raman spectroscopy by using Horiba iH320 spectrometer with a 532 nm laser source, X-ray diffraction (XRD) was acquired by using a Mo-Kα source (λ = 0.709319 Å) coupled with a curved multi-channel detector INEL CPSD 180 (Curved Position Sensitive Detector). For the sake of comparison with literature findings, the reflection angle values were converted into 2*θ*/Cu Kα. Thermal analysis of Fe_3_O_4_ composite was carried out by employing a TGA-DTA Perkin-Elmer STA6000. The morphological characterization was performed with a ZEISS Sigma Series 300 field emission scanning electron microscope (FE-SEM).

### 3.5. Cell Assembly and Electrochemical Test

Three-electrode Swagelok-type cells with metallic lithium foil as counter and reference electrode, stainless steel current collectors were employed for characterization of electrochemical performances. Circular Whatman GF/A glass fiber 12 mm diameter disks were used as separator and 1 M LiPF_6_ in Ethylene carbonate (EC):Dimetheyl carbonate (DMC) 1:1 (v:v) (Solvionic, Tolouse, France) was used as electrolyte. 

The electrochemical behavior of the electrodes was characterized by a VMP-3 galvanostat/potentiostat (Bio-Logic). Cyclic voltammetry (CV) was acquired in the potential range within 0.001–3.000 V at 0.1 mV s^−1^ scan rate. GCPL cycles were performed in the same potential range and at C-rates between C/10 (calculated as 88.1 mA g^−1^) and 10 C (8810 mA g^−1^). Electrochemical impedance spectroscopy (EIS) measurements were performed at bias potential E = 0.75 V for the first cycle and E = 0.90 V for the subsequent cycles, by applying a sinusoidal perturbation of amplitude ΔE = ±5 mV in the frequency range 101 kHz < f < 9 mHz. All potential values are given vs. Li^+^/Li redox couple (E°_Li+/Li_ = −3.04 V vs. SHE). 

## 4. Conclusions

Fe_3_O_4_ nanoparticles were synthesized by a base-promoted method and characterized, resulting in an average nanoparticle size of about 6 nm computed by Scherrer’s equation and confirmed by SEM micrograph. 

A composite material based on Fe_3_O_4_ nanoparticles and reduced graphene oxide was synthesized by ultrasonication of graphene oxide and subsequent reduction with hydrazine. The synthesized composite was characterized, giving an average nanoparticle size of 6 nm and confirming the embedding of magnetite by graphene matrix. The electrochemical tests revealed a quite stable charge/discharge behavior of the composite (~900 mAh g^−^^1^ at 1C-rate after the rate stresses), and impressive rate capability (980 mAh g^−1^ at 4C; 484 mAh g^−1^ at 10C). The EIS measurements confirmed that the outstanding cycling performances are rooted in stable morphology and low charge-transfer polarization, given by the embedding of nanoparticles into reduced graphene oxide sheet.

The combination of low-environmental-impact and facility of the synthesis of the composite material and electrode processing, together with the high performances herein reported, make the composite a promising anode material for high-energy and especially high-power applications.

## Figures and Tables

**Table 1:** Specific capacities and efficiencies of the Fe_3_O_4_/rGO at different C-rates, as from Figure 7a.

	Cycle Number	C-Rate	Capacity/mAh g^−1^	Efficiency/%
A	3	C/10	1253	88.6
B	8	C/5	1069	93.6
C	13	C/2	925	96.4
D	18	1C	834	97.7
E	23	2C	742	98.0
F	28	5C	610	99.1
G	33	10C	484	99.2
H	50	1C	874	99.6

**Table 2:** Comparison of low- and high-C-rate performances of Fe_3_O_4_-based composite anodes.

	Low C-Rate		High C-Rate		
Electrode	Current/A g^−1^	Capacity/mAh g^−1^ (Cycle Number)	Current/A g^−1^	Capacity/mAh g^−1^ (Cycle Number)	Ref.
Fe_3_O_4_@rGO	1	1260 (250)	10	357 (65)	[37]
C-Fe_3_O_4_	0.2	1065 (200)	8	470 (50)	[38]
CNT-Fe_3_O_4_@graphene	0.2 C *	≈600 (5)	10 C *	177 (25)	[39]
Fe_3_O_4_@rGNSs-CNTs	0.1	1232.9 (100)	5	500 (30)	[40]
Fe_3_O_4_/rGO	0.88	688 (100)	8.81	484 (33)	This work

* No specific current values available in the cited manuscript.

## Data Availability

Data available on request.
